# The role of dysfunctional attitudes towards motherhood in postpartum depressive symptoms and disorder

**DOI:** 10.1192/j.eurpsy.2021.480

**Published:** 2021-08-13

**Authors:** D. Pereira, C. Cabaços, J. Azevedo, S. Xavier, M.J. Soares, N. Madeira, A. Macedo, A.T. Pereira

**Affiliations:** 1 Institute Of Psychological Medicine, Faculty Of Medicine, University of Coimbra, coimbra, Portugal; 2 Psychiatry Department, Centro Hospitalar e Universitário de Coimbra, Coimbra, Portugal; 3 Institute Of Psychological Medicine, Faculty Of Medicine, University of Coimbra, Coimbra, Portugal

**Keywords:** dysfunctional attitudes towards motherhood, perinatal, postpartum depressive disorder

## Abstract

**Introduction:**

Postpartum depression (PPD) is the commonest postpartum psychiatric condition, with prevalence rates around 20%^1^. PPD is associated with a range of adverse outcomes for both the mother and infant^2^. Therefore, identifying modifiable risk factors for perinatal depression is an important public health issue^3^.

**Objectives:**

To explore the role of dysfunctional attitudes towards motherhood in postpartum depressive symptoms and disorder.

**Methods:**

247 women were evaluated in the third (12.08±4.25 weeks) and sixth months (31.52± 7.16 weeks) postpartum with the Attitudes Towards Motherhood Scale^4^, the Postpartum Depression Screening Scale^5^ and the Diagnostic Interview for Psychological Distress-Postpartum^6^. Correlation analysis was performed followed by linear/logistic regression analysis when the coefficients proved significant (p<.05), using SPSS.

**Results:**

Dysfunctional beliefs towards motherhood concerning judgement by others and maternal responsibility positively correlated with depressive symptoms at the third (.528; .406) and the sixth months (.506; .492) postpartum. Those dysfunctional beliefs were predictors of depressive symptoms at the third (ß=.440; ß=.151) and sixth months (ß=.322; ß.241) explaining 29.4% and 30.2% of its variance, respectively. Having dysfunctional beliefs at the third month significantly increase the likelihood of being diagnosed with Major Depression (DSM5) both in the third (Wald=9.992, OR=1.169; Wald=16.729, OR=1.231) and sixth months (Wald=5.638, OR=1.203; Wald=7.638, OR=1.301) (all p<.01).

**Conclusions:**

Cognitive distortions should be included in the assessment of risk factors for PPD. Early identification of women presenting motherhood-specific cognitive biases may be crucial for implementing preventive interventions favoring a more positive and healthier motherhood experience.

**Disclosure:**

No significant relationships.

